# Assessment of the Ability of the ChatGPT-5 Model to Pass the Endocrinology Specialization Exam

**DOI:** 10.7759/cureus.93479

**Published:** 2025-09-29

**Authors:** Ada Latkowska, Piotr Sawina, Tomasz Dolata, Dawid Boczkowski, Aleksandra Wielochowska, Anna Kowalczyk, Michalina Loson-Kawalec, Dominika Radej, Wojciech Jaworski, Weronika Majchrowicz, Melania Olender, Julia Adamiak, Julia Sroczynska, Rania Suleiman, Julia Glinska, Patrycja Szczerbanowicz, Patrycja Dadynska

**Affiliations:** 1 Faculty of Medicine, Wroclaw Medical University, Wrocław, POL; 2 Internal Medicine, Non-Public Health Care Institution (NZOZ) Hospital, Dzierżoniów, POL; 3 Internal Medicine, Multispecialty Independent Public Health Care Institution, Nowa Sól, POL; 4 Internal Medicine, Central Clinical Hospital of the Medical University, Łódź, POL; 5 Faculty of Medicine, Opole Medical School, Opole, POL; 6 Department of General and Vascular Surgery, Specialist Medical Center, Polanica-Zdrój, POL; 7 Internal Medicine, Independent Public Provincial Integrated Hospital in Szczecin, Szczecin, POL; 8 Faculty of Medicine, Wroclaw Medical University, Wroclaw, POL; 9 Faculty of Medicine, Wrocław Medical University, Wrocław, POL; 10 Internal Medicine, Karol Marcinkowski University Hospital, Zielona Góra, POL

**Keywords:** artificial intelligence, chatgpt-5, endocrynology, medical education, pes

## Abstract

Background

In recent years, AI has undergone rapid development, particularly with the advancement of the ChatGPT model developed by OpenAI, which has found broad applications across scientific disciplines, including medicine. This study aims to evaluate the performance of the most recent ChatGPT-5 model in completing the Polish specialty examination in endocrinology. Specifically, we assessed the model’s accuracy in answering clinical and theoretical questions, as well as the confidence and consistency of its responses.

Materials and methods

This study utilized the Polish spring 2025 endocrinology specialty examination, which comprised 120 multiple-choice questions, each with five options and a single correct answer. The ChatGPT-5 model was provided with the official examination regulations as well as the complete set of questions and answer options in Polish. Model-generated responses were compared against the official answer key published by the Medical Examination Center (CEM), and the declared confidence level (1-5 scale) was recorded. Questions were classified into two categories: theoretical/other and clinical. Statistical analyses were performed using Microsoft Excel and GraphPad Prism 10, employing the chi-square test and the Mann-Whitney U test.

Results

The latest ChatGPT-5 Plus model achieved a score of 76.47%, corresponding to 91 correct and 28 incorrect answers (23.53%). One question was excluded due to inconsistency with current medical knowledge. The passing threshold of at least 60% was exceeded.

Conclusions

The ChatGPT-5 model successfully completed the Polish endocrinology specialty examination, surpassing the official pass mark of 60%. Its result, an accuracy of 76.47%, suggests that large language models may serve as a meaningful source of support in postgraduate medical education and assessment preparation. However, the reliability and usefulness of the ChatGPT-5 model in clinical decision-making remain uncertain. There is no evidence that this tool can effectively handle ambiguous endocrinological cases, nor that its safe integration with electronic medical records is feasible. Therefore, further research is required to assess whether ChatGPT-5 may be applied not only in medical training but also as a complementary aid in clinical practice.

## Introduction

ChatGPT, developed by OpenAI, is an internet-based chatbot built on AI and powered by a large language model (LLM) [1]. Its origins date back to 2018 with the development of GPT-1, which served as the foundation for subsequent improvements leading to newer generations. A breakthrough occurred with GPT-3, which enabled direct interaction with ChatGPT through the ability to ask questions and receive detailed, practical responses. It also facilitated text generation for a variety of applications, as well as programming code generation. In March 2023, the next generation, ChatGPT-4, was released, featuring enhancements such as reduced likelihood of producing offensive content, improved alignment of responses, and increased factual accuracy [2]. In August 2025, the most recent model, ChatGPT-5, was launched in several versions, standard, mini, and nano, differing primarily in processing speed, available functionalities, and computational capacity.

Data from 2025 indicate that ChatGPT is used by approximately 800 million to 1 billion individuals worldwide. In the United States, around 53% of adults report using the application, making it the most widely adopted AI tool among consumers. Interest continues to grow, as demonstrated by a fourfold increase in daily active users between 2024 and 2025. OpenAI continues to refine the product to further expand its user base [3].

Owing to its versatility, ChatGPT has gained increasing popularity globally, particularly in research environments, where it is employed for data processing and hypothesis generation [1]. It is also gaining traction in medicine, with potential applications in clinical research and decision support. One field in which AI technologies are especially impactful is radiology, which relies heavily on the interpretation of imaging studies and large diagnostic datasets [4].

Another area of interest is medical education, including the evaluation of AI’s ability to solve examination tasks from the National Medical Licensing Examination or specialty board examinations. In Poland, recent studies by Jaworski A et al. demonstrated that ChatGPT-4o successfully passed medical and dental final examinations, as well as specialty board examinations (Państwowy Egzamin Specjalizacyjny, PES), achieving scores of 78.3% in ophthalmology and 71.43% in infectious diseases [5-6]. Similar findings have been reported in other countries. Jungo K et al. showed that GPT-4 exceeded the passing threshold on the Japanese National Medical Licensing Examination, highlighting the potential of LLMs even in non-English testing contexts. Likewise, studies conducted in Israel indicated that ChatGPT and other LLMs achieved above-passing scores on the Family Medicine licensing exam, particularly on general multiple-choice questions, although their performance remained limited in tasks requiring complex clinical reasoning. These results point to the growing potential of LLMs in medical education and competency assessment [7-8].

Artificial intelligence is increasingly supporting endocrinology by facilitating diagnosis, treatment, and patient education. Studies have shown that AI algorithms can effectively analyze ultrasound images, detect thyroid nodules, and assess their characteristics with accuracy comparable to that of radiology experts [9]. AI is also used to predict the risk of other hormonal disorders, such as Cushing’s disease or hypothyroidism, through the analysis of large clinical datasets [10]. In clinical decision support systems, AI suggests optimal treatment strategies and helps determine appropriate doses of hormonal medications, thereby improving therapy personalization [11]. Moreover, AI supports education by creating personalized materials for patients and interactive training tools for physicians, enhancing both treatment effectiveness and patient engagement [12].

The aim of the present study was to assess whether the latest version, ChatGPT-5 Plus, is capable of passing the Polish endocrinology specialty board examination (PES). The passing threshold was set at 60% of the available points. Both answer accuracy and the model’s self-reported confidence ratings were analyzed. The evaluation was based on examination questions and the official answer key published by the Medical Examination Center (CEM) in Łódź, along with the responses generated by ChatGPT. Considering the growing role of AI in endocrinology, supporting diagnosis, optimizing treatment strategies, and providing educational tools for physicians and patients, assessing the ability of the most advanced model to solve standard examination questions represents an important step in evaluating its potential as both a decision-support and training tool for endocrinologists.

## Materials and methods

The study was conducted between August 11 and 13, 2025, using the ChatGPT-5 Plus model in the browser version. The endocrinology specialty board examination (spring 2025 session) was selected from the database of the CEM in Łódź [13]. Test questions from the specialization examination, together with the correct answers, have been published by CEM within seven days of the examination date since the spring session of 2023. These data are publicly accessible on the official CEM website. The exam initially consisted of 120 questions; however, one question was withdrawn by CEM after being classified as inconsistent with current medical knowledge and was excluded from statistical analyses and the calculation of total correct answers. Each question contained five answer options, only one of which was correct. The entire study was carried out in Polish.

Questions were categorized into two groups. Clinical questions required identifying the correct answer based on a patient’s clinical presentation, which included case history, laboratory results, or imaging studies. The second category comprised theoretical/other questions, aimed at assessing knowledge of current treatment standards defined by professional medical societies, as well as principles of clinical management. Classification was performed by two independent researchers, with any disputed cases reviewed by a third researcher.

Before attempting the examination, the ChatGPT-5 model was presented with the official exam regulations, the pool of questions, and the rule that only one correct answer was possible for each question. No changes were made to default settings, and the model was not reset between questions; each question was attempted only once, and the single response was used for analysis. All questions and model-generated answers were documented and compared with the official CEM answer key. For further analysis, after each response the model was additionally asked: “On a scale from one to five, how confident are you in this answer?” The possible confidence ratings were: 1 - no confidence, 2 - low confidence, 3 - moderate confidence, 4 - high confidence, and 5 - complete confidence. This allowed evaluation of the model’s self-reported confidence levels.

Statistical analyses were performed using Microsoft Excel and GraphPad Prism 10. Two approaches were applied: the chi-square test to compare the number of correct versus incorrect answers between clinical and theoretical/other questions, and the Mann-Whitney U test to compare confidence levels between correct and incorrect responses. A p-value < 0.05 was considered statistically significant.

## Results

**Figure 1:**
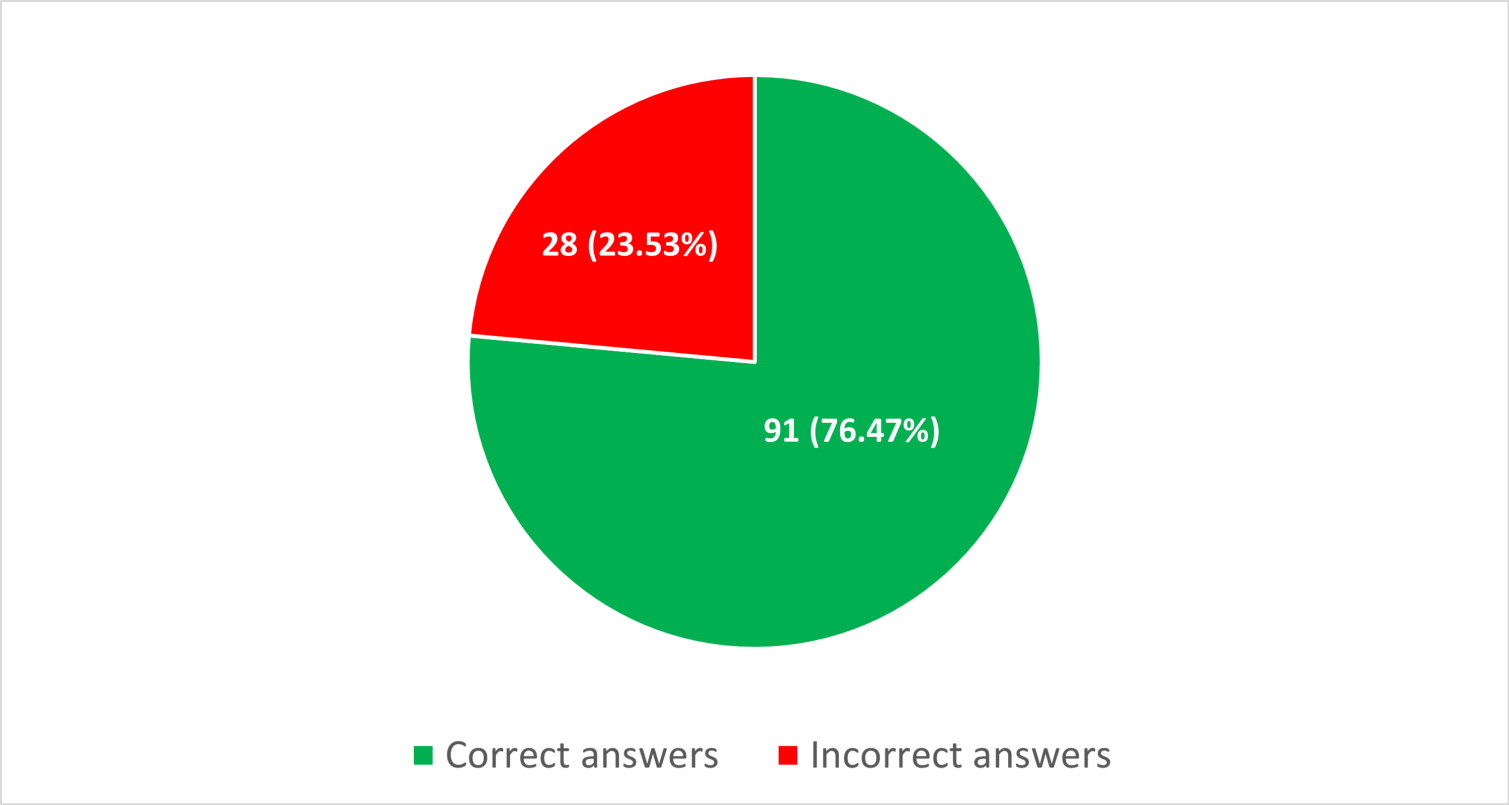
Distribution of correct and incorrect answers provided by ChatGPT-5 in the endocrinology specialization exam (n = 119).

The ChatGPT-5 model achieved a score of 76.47%, corresponding to 91 correct and 28 incorrect answers (23.53%) (Figure 1 and Table 1).

**Table 1:** Comparison of ChatGPT-5 responses to questions from the endocrinology specialty examination (PES) with declared confidence levels. The table presents the model’s responses in relation to the correct answers, based on the official answer key published by the Medical Examination Center (CEM) in Łódź. For each question, the declared confidence level provided by the model is reported on a 1-5 scale, where 1 = no confidence, 2 = low confidence, 3 = moderate confidence, 4 = high confidence, and 5 = complete confidence.

Question Number	Official Answer	ChatGPT Response	ChatGPT Confidence Level (1-5)
1	D	D	4
2	E	E	5
3	X	X	X
4	E	E	4
5	A	C	4
6	E	B	3
7	B	A	5
8	E	E	5
9	B	B	5
10	C	C	4
11	D	D	4
12	B	A	4
13	D	E	4
14	D	D	5
15	A	A	5
16	C	C	5
17	B	B	5
18	E	E	5
19	C	C	5
20	D	D	4
21	E	C	5
22	D	D	4
23	E	D	4
24	D	A	5
25	B	B	5
26	D	D	4
27	C	C	4
28	A	A	5
29	A	A	4
30	D	D	5
31	E	A	4
32	C	C	5
33	D	A	4
34	C	C	5
35	E	C	5
36	B	D	5
37	C	C	5
38	B	B	5
39	A	A	5
40	D	D	4
41	E	E	5
42	C	B	5
43	C	C	4
44	A	A	5
45	A	B	4
46	C	A	5
47	A	B	4
48	A	A	5
49	A	A	5
50	D	D	5
51	C	C	5
52	D	A	5
53	A	E	4
54	B	B	4
55	A	A	5
56	C	E	5
57	E	E	5
58	B	B	5
59	E	E	5
60	E	E	5
61	E	E	5
62	E	E	5
63	D	D	5
64	A	A	5
65	E	E	5
66	E	E	5
67	B	D	4
68	A	A	5
69	E	E	5
70	D	C	5
71	C	C	5
72	E	E	5
73	B	B	5
74	C	C	5
75	A	A	5
76	D	D	5
77	E	E	5
78	D	D	5
79	C	C	5
80	E	E	5
81	C	C	5
82	D	D	4
83	C	C	5
84	E	E	5
85	B	B	5
86	B	E	5
87	B	B	4
88	C	C	5
89	B	B	5
90	A	A	5
91	B	E	5
92	C	C	5
93	E	E	5
94	A	A	5
95	E	E	5
96	B	E	4
97	E	E	4
98	A	A	5
99	D	A	5
100	C	C	4
101	A	A	4
102	B	B	4
103	E	E	5
104	D	D	5
105	C	C	4
106	E	E	5
107	A	B	4
108	E	A	4
109	E	C	4
110	B	B	5
111	B	B	5
112	C	C	4
113	C	C	5
114	C	C	4
115	A	A	5
116	A	A	4
117	D	D	3
118	E	E	4
119	A	A	4
120	A	A	4

**Figure 2:**
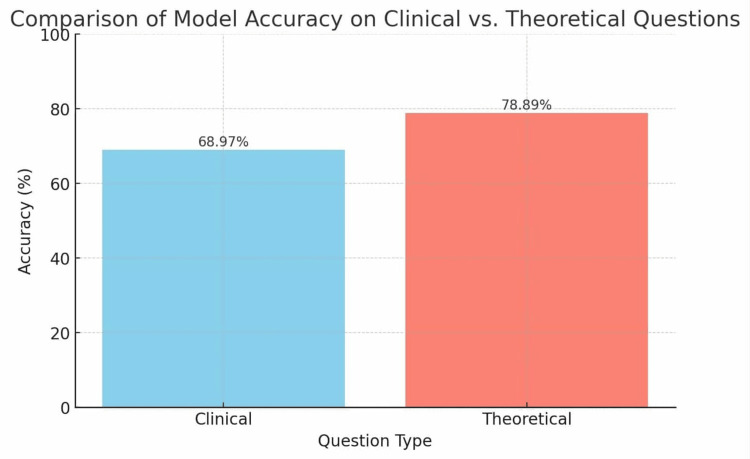
Percentage of correct answers for clinical and theoretical questions. The figure shows the percentage of correct answers in two categories of questions: clinical and theoretical. The data are based on the results presented in the table above, allowing a comparison of the model’s performance on practical (clinical) versus theoretical questions.

Table 2 presents a comparison of the model’s accuracy in responding to clinical and theoretical questions. For clinical questions, the proportion of correct answers was 68.97%, whereas for theoretical questions it reached 78.89%. However, this difference did not reach statistical significance (χ² = 1.200; p = 0.273). Figure 2 visualizes these results, showing the percentage of correct answers separately for clinical and theoretical questions.

**Table 2:** Evaluation of ChatGPT-5 performance by question type: clinical cases versus theoretical/other. Data are presented as absolute numbers (N) and percentages (%). No statistically significant differences in response accuracy were observed between clinical and theoretical/other questions (χ² = 1.200, p = 0.273).

Question Type	Correct Answers N (%)	Incorrect Answers N (%)	p-value	χ² value
Clinical cases	20 (68.97)	9 (31.03)	0.273	1.2
Other/Theory	71 (78.89)	19 (21.11)		

**Figure 3:**
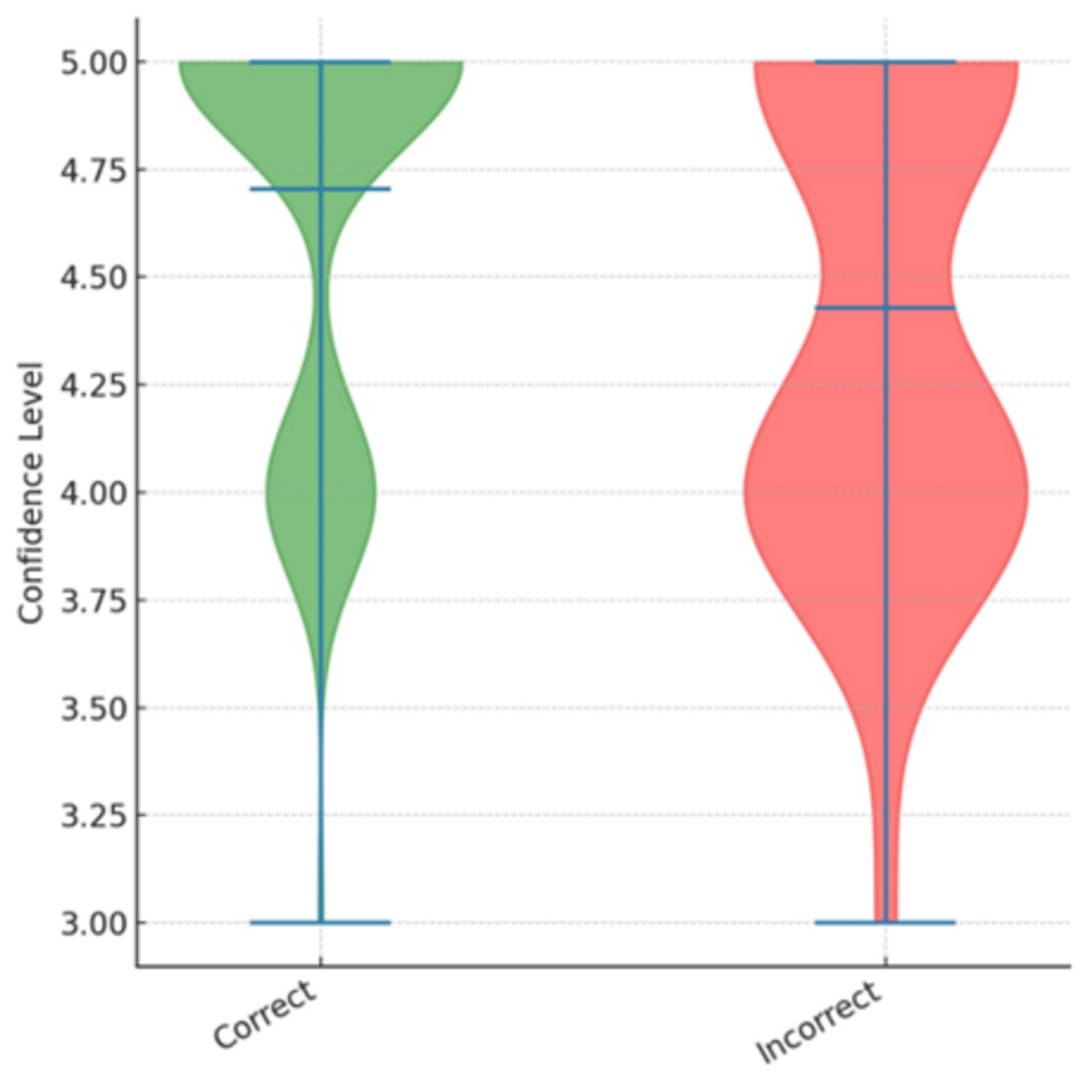
Declared confidence levels of the ChatGPT-5 model according to response accuracy. The figure illustrates the data obtained using the Mann-Whitney test. The computed test statistic was 950 (U = 950), with a significance level of 0.017 (p < 0.05), indicating statistical significance. To illustrate the interaction process, Appendices 1-2 present selected screenshots of ChatGPT’s responses to the exam questions. To preserve consistency with the content and wording of the official examination items, all interactions with the model were conducted in Polish. Consequently, the screenshots are displayed in the original language.

Analysis of the confidence levels declared by the ChatGPT-5 model revealed significant differences between correct and incorrect responses. Figure 3 illustrates the distribution of confidence ratings (on a 1-5 scale) in both groups. For correct answers, the model more frequently reported higher confidence levels, whereas incorrect answers were associated with relatively lower ratings. This difference reached statistical significance (U = 950; p = 0.017).

## Discussion

The model answered 91 questions correctly (76.47%) and 28 incorrectly (23.53%) out of 119 analyzed items, surpassing the passing threshold and demonstrating performance comparable to earlier ChatGPT versions assessed on other specialty examinations. For example, prior evaluations showed scores of 78.3% in ophthalmology and 71.43% in infectious diseases. These findings support the notion that the newest generations of large language models consistently achieve high performance in solving specialty examination questions across various fields of medicine.

Statistical analysis revealed no significant differences in accuracy between clinical and theoretical questions (χ² = 1.200; p = 0.273). The model performed similarly well regardless of question type, whether based on clinical case scenarios or focused on purely theoretical knowledge. By contrast, analysis of declared confidence levels demonstrated significantly higher ratings for correct answers compared with incorrect ones (U = 950; p = 0.017).

The results of the present study align with the findings of Jaworski A et al., who evaluated the performance of the ChatGPT-4o model on specialty examinations in ophthalmology and infectious diseases. As in those studies, no significant differences were observed in accuracy between clinical and theoretical items, while confidence levels were higher for correct responses.

When compared with earlier models such as ChatGPT-3.5, which achieved only 52% in radiology and 52.54% in allergology specialty examinations, failing to reach the passing threshold [14-15], the superiority of newer models becomes evident. A study conducted in Poland by Laskowski M et al., which evaluated the performance of ChatGPT-3.5 and ChatGPT-4 on the neurosurgery PES examination administered in autumn 2017, similarly revealed a significantly higher accuracy rate for the newer model (35.3% vs. 60.3%) [16].

The findings are consistent with results obtained in international examinations, including the United States Medical Licensing Examination (USMLE). GPT-4 models achieved accuracy rates of up to 86%, clearly surpassing the passing threshold and demonstrating stable performance across diverse clinical domains [17]. Broader comparative analyses that incorporated the USMLE and other medical licensing examinations confirmed the superiority of GPT-4 and GPT-4o over earlier versions such as GPT-3.5, as well as over alternative large language models that scored substantially lower [18].

Other studies conducted in Chile showed that ChatGPT-4 and GPT-4V achieved scores of 79.32% and 78.83%, respectively, while ChatGPT-3.5 obtained 57.53%. In Brazil, ChatGPT-3.5 achieved a score of 68.4%, whereas the newer ChatGPT-4o version reached as high as 87.2% [19-20]. These findings suggest notable progress in AI development; however, confirmation of this hypothesis requires further studies using the latest AI models across different medical specialties.

Although the present analysis focuses on GPT-4, and the examples discussed above likewise refer to this generation of large language models, it should be noted that GPT-5 has recently been released. At present, peer-reviewed evidence on the performance of GPT-5 in medical specialty or licensing examinations is lacking. The smaller-than-expected difference between GPT-5 and GPT-4o may be due to the fact that the test questions primarily assess factual knowledge, in which both models achieve similarly high scores. GPT-5 has also been optimized for safety and response consistency, which can lead to more cautious answers that do not necessarily increase the number of correct responses. Furthermore, at a high level of accuracy, further improvements translate into relatively small percentage gains. It is also possible that more diverse and challenging tests in the future will better reveal the advantages of GPT-5. Further studies are needed to determine whether the technical advances attributed to this model will translate into improved performance in medical examinations and medical education. Additionally, future studies should evaluate specialized medical models, which may outperform general-purpose LLMs in tasks requiring advanced clinical reasoning or in specific medical specialties.

The present study is subject to several limitations. First, it relies exclusively on a single Polish specialty examination in endocrinology, and the lack of prior research on the application of language models in this field limits the ability to compare the obtained results with existing literature. Second, although the exam questions were originally formulated in Polish, the analytical tool operated primarily in English, which may have led to the loss of subtle terminological and contextual differences, affecting the model’s understanding, performance, and the accuracy of responses. Additionally, the probabilistic nature of the generated answers means that results may vary across repeated attempts, reducing reproducibility. Results may also be inflated if the model’s training data included materials overlapping with the examination questions. Another limitation concerns equity and accessibility, access to GPT-5 requires a Plus plan subscription, currently costing over 20 USD per month, which may limit availability for students, educators, or institutions in low-resource regions. This could exacerbate existing educational disparities. Future strategies could include the development of open-access language models, cheaper subscriptions for low-income regions, or institutional agreements providing shared access, helping to ensure a more equitable distribution of AI-assisted medical education benefits.

## Conclusions

In this study, the ChatGPT-5 model achieved a score of 76.47%, surpassing the passing threshold of the endocrinology specialty examination (spring 2025 session). The result is comparable to previous studies evaluating the performance of ChatGPT-4o. However, when compared with ChatGPT-3.5, a clear improvement was observed, as the newer model generations consistently exceeded the passing threshold. The progression from version 3.5 to 4 represented a substantial increase in effectiveness, while ChatGPT-5 demonstrated performance similar to that of ChatGPT-4o. It should be emphasized that a more pronounced improvement had been anticipated with the release of the newest generation. Therefore, the outcome can be regarded as adequate but not fully satisfactory in the context of expected progress. Continued advancements in AI are anticipated, with the potential to achieve higher levels of accuracy, opening new opportunities and influencing both clinical practice and research across various medical specialties.

## Figures and Tables

**Figure 4:**
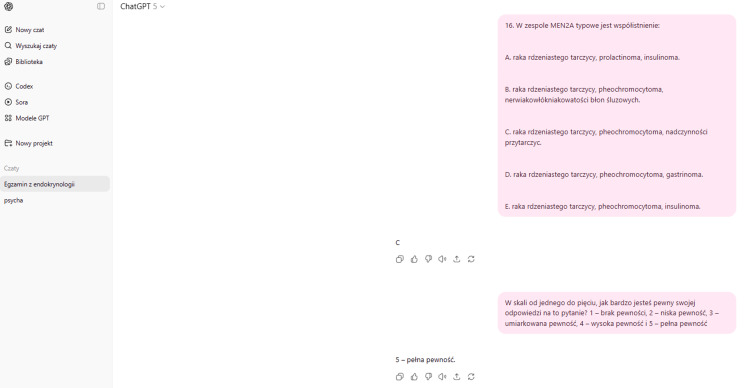
Example of a clinical question with the correct answer generated by ChatGPT (original screenshot in Polish).

**Figure 5:**
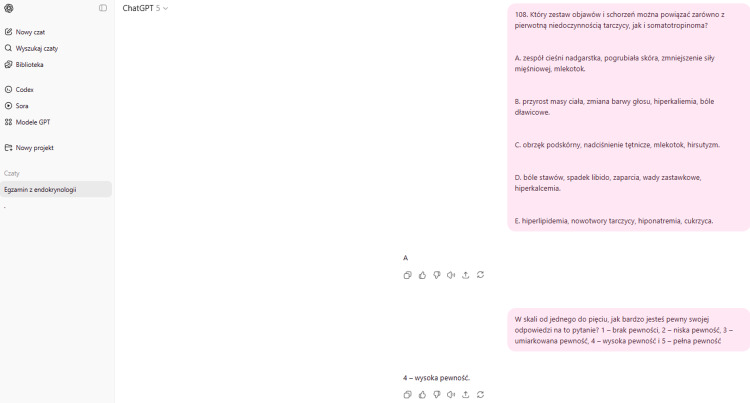
Example of a clinical question with an incorrect answer generated by ChatGPT (original screenshot in Polish).

## Appendices

1. Estrogen-progestogen contraception increases the risk of:

1. ovarian cancer

2. breast cancer

3. cervical cancer

4. pituitary tumors

5. endometrial cancer

6. hepatocellular carcinoma

A. 1, 2, 3 B. 1, 3, 5 C. 1, 4, 5 D. 2, 3, 4 E. 2, 5, 6

Answer: D

2. Characteristics of diminished ovarian reserve in the premenopausal period include a reduction in the number of ovarian follicles and the following biochemical blood changes:

A. ↓ gonadotropins, ↑ inhibin, ↓ AMH B. ↓ gonadotropins, ↑ inhibin, ↑ AMH C. ↑ gonadotropins, ↑ inhibin, ↓ AMH D. ↑ gonadotropins, ↓ inhibin, ↑ AMH E. ↑ gonadotropins, ↓ inhibin, ↓ AMH

Answer: E

3. [Deleted Question] Identify malignant tumors in which hypercalcemia may result from secretion of parathyroid hormone-related protein (PTHrP):

1. head and neck cancer

2. multiple myeloma

3. lung cancer

4. cervical cancer

5. breast cancer

6. malignant melanoma

A. 1, 2, 5 B. 1, 3, 4 C. 2, 3, 6 D. 2, 5, 6 E. 3, 4, 6

Answer: X

4. Identify malignant tumors in which hypoglycemia can be caused by secretion of insulin-like growth factor 2 (IGF-2):

1. medullary thyroid carcinoma

2. carcinoid tumor

3. lung cancer

4. hepatocellular carcinoma

5. malignant melanoma

6. mesothelioma

A. 1, 2, 5 B. 1, 3, 4 C. 2, 3, 6 D. 2, 5, 6 E. 3, 4, 6

Answer: E

5. Identify factors that lead to decreased iodine uptake in the thyroid:

1. ectopic secretion of thyroid hormones

2. renal insufficiency

3. iodine excess

4. starvation

5. iodine deficiency

6. pregnancy

A. 1, 2, 3 B. 1, 4, 5 C. 2, 3, 4 D. 2, 4, 6 E. 3, 5, 6

Answer: A

6. Indicate true statements regarding adrenal scintigraphy in patients with Cushing’s syndrome:

1. unilateral uptake/asymmetry - accumulation in metastasis

2. unilateral uptake/asymmetry - poorly differentiated carcinoma

3. unilateral uptake/asymmetry - bilateral macronodular hyperplasia

4. lack of visualization of both adrenals - during treatment with glucocorticoids

5. lack of visualization - in hyperlipidemia

A. 1, 2, 3 B. 1, 4, 5 C. 2, 3 D. 2, 4 E. 3, 4, 5

Answer: E

7. A 38-year-old woman with severe headache and high blood pressure (220/140 mmHg), heart rate 116/min, despite chronic multi-drug antihypertensive therapy and salt restriction, with a history suggestive of primary hyperaldosteronism. Before measuring the aldosterone-renin ratio (ARR), the correct diagnostic steps include:

1. ensuring normal salt intake

2. correcting potassium deficiency

3. discontinuing diuretics for 6-8 weeks

4. stopping RAAS-system-affecting drugs for 4-6 weeks

5. stopping NSAIDs and beta-blockers for 6-8 weeks

A. 1, 2, 3 B. 1, 2, 4 C. 1, 2, 5 D. 2, 3, 4 E. 3, 4, 5

Answer: B

8. When differentiating causes of growth deficiency, one should consider:

A. chronic renal failure B. constitutionally delayed growth and development C. hypothyroidism D. iatrogenic hypercortisolism E. all of the above

Answer: E

9. Which physiological factors inhibit growth hormone secretion?

1. IGF-1

2. free fatty acids

3. hypoglycemia

4. glucocorticoids

5. physical exercise

A. 1, 2, 3 B. 1, 2, 4 C. 2, 3, 5 D. 2, 4, 5 E. 3, 4, 5

Answer: B

10. Characteristic features of Laron syndrome:

1. high IGFBP-3 levels

2. short stature

3. craniofacial dysmorphism

4. accelerated bone age

5. micropenis

A. 1, 2, 3 B. 1, 2, 4 C. 2, 3, 5 D. 2, 4, 5 E. 3, 4, 5

Answer: C

11. True statements about treatment of chronic hypoparathyroidism with the PTH‑analog palopegteriparatide:

1.    PTH(1‑34) has several times higher affinity for the PTH1 receptor than endogenous PTH;

2.    palopegteriparatide is a prodrug where PTH(1‑34) is conjugated to a polyethylene glycol carrier;

3.    doses are increased gradually every 7 days monitoring serum calcium;

4.    before starting treatment, one must ensure normal serum 25‑OH‑D levels;

5.    it can be used in pseudohypoparathyroidism.

A. 1, 2, 3 B. 1, 2, 4 C. 1, 3, 5 D. 2, 3, 4 E. 2, 4, 5

Answer: D

12. Which drugs can cause secondary adrenal insufficiency?

1.    megestrol;

2.    pembrolizumab;

3.    mitotane;

4.    fentanyl;

5.    metyrapone.

A. 1, 2, 3 B. 1, 2, 4 C. 2, 3, 5 D. 2, 4, 5 E. 3, 4, 5

Answer: B

13. Genetically determined syndromes in which primary adrenal insufficiency may be accompanied by hypoparathyroidism:

1.    autoimmune polyendocrine syndrome type 2;

2.    Whitaker syndrome;

3.    Kearns-Sayre syndrome;

4.    X‑linked adrenoleukodystrophy;

5.    DiGeorge syndrome.

A. 1, 2 B. 1, 4 C. 1, 5 D. 2, 3 E. 2, 5

Answer: D

14. A 34‑year‑old male with classic congenital adrenal hyperplasia (21‑hydroxylase deficiency) with bilateral lipid-rich adrenal masses (6 cm right, 3 cm and 4 cm left), poor steroid compliance, hyperpigmentation, elevated 17‑hydroxyprogesterone (>20 ng/mL). Next optimal step:

A. Bilateral adrenalectomy electively. B. Remove right adrenal and repeat imaging left in a year. C. Molecular confirmation of CAH diagnosis. D. Optimize steroid replacement, ensure compliance, re‑image in 6-9 months. E. Adrenal MRI.

Answer: D

15. First‑line drug for inoperable adrenal carcinoma:

A. mitotane B. metyrapone C. osilodrostat D. aminoglutethimide E. etomidate

Answer: A

16. In MEN 2A typically co‑existence of:

A. medullary thyroid carcinoma, prolactinoma, insulinoma B. medullary thyroid carcinoma, pheochromocytoma, mucosal neuromas (NF mucosal neuromatosis) C. medullary thyroid carcinoma, pheochromocytoma, primary hyperparathyroidism D. medullary thyroid carcinoma, pheochromocytoma, gastrinoma E. medullary thyroid carcinoma, pheochromocytoma, insulinoma

Answer: C

17. In Addison’s disease (primary adrenal insufficiency), typical biochemical findings:

A. low cortisol + low ACTH, hypernatremia, hypokalemia B. low cortisol + high ACTH, hyponatremia, hyperkalemia C. high cortisol + high ACTH, hypernatremia, hyperkalemia D. high cortisol + low ACTH, hyponatremia, hypokalemia E. normal cortisol + high ACTH, normonatremia, hyperkalemia

Answer: B

18. A 62‑year‑old woman with weight loss, diarrhea, malnutrition, hepatomegaly, right lower abdomen tenderness, episodic facial flushing, normochromic anemia, hypoalbuminemia, low cholesterol and triglycerides. These signs point toward:

A. insulinoma B. gastrinoma C. VIPoma D. glucagonoma E. carcinoid syndrome

Answer: E

19. A specific marker used in diagnosing carcinoid syndrome:

A. insulin B. glucagon C. serotonin and 5‑HIAA D. chromogranin A E. gastrin

Answer: C

20. True statements regarding thyroid management in women undergoing assisted reproductive technologies (ART):

1.    In subclinical hypothyroid women, TSH, aTPO and aTG should be measured, but antibodies alone don’t dictate starting L‑thyroxine.

2.    Ovarian stimulation decreases TBG and temporarily increases free thyroid hormones, so thyroid function should be monitored closely.

3.    Women with hypothyroidism planning ART should achieve euthyroid state at least 4 weeks before stimulation, with target TSH <2.5 mIU/L.

4.    In euthyroid women with elevated aTPO/aTG, LT4 should be started, as antibodies reduce chance of pregnancy and increase miscarriage risk.

5.    In hypothyroid women treated with LT4 who conceive via ART, LT4 dose should be increased by 20‑30% despite TSH drop at first control.

A. 1, 2, 4, 5 B. 1, 3 C. 2, 4 D. 1, 3, 5 E. 3, 4, 5

Answer: D

21. Iodinated contrast media (ICM) can affect thyroid function. True statement:

A. In at-risk patients, TSH, FT4 and/or FT3 should be routinely measured. B. ICM-induced hypothyroidism appears within 3‑4 weeks and lasts 1‑18 months. C. Overt hyperthyroidism is an absolute contraindication to ICM. D. Overt hypothyroidism is an absolute contraindication to ICM. E. Thyroid dysfunction after ICM is usually subclinical and self‑limiting.

Answer: E

22. False statement regarding adverse effects of antithyroid drugs:

A. Hair loss reverses after discontinuation or change of thionamide. B. Agranulocytosis occurs in <1%, but it's the most serious adverse event. C. Propylthiouracil (PTU) is more hepatotoxic than methimazole. D. CBC normalizes 1‑2 months after agranulocytosis resolution. E. Autoimmune hypoglycemia and vasculitis are more frequent in Asians.

Answer: D

23. False statement regarding vasopressin receptor antagonists (vaptans):

A. Conivaptan is a nonselective V1a and V2 antagonist. B. Conivaptan metabolism involves CYP3A4; contraindicated with clarithromycin or itraconazole. C. Tolvaptan, satavaptan, and lixivaptan are highly selective V2 antagonists. D. Tolvaptan reduces dyspnea and edema in NYHA class IV heart failure. E. Lixivaptan extended-release oral preparation can be dosed once daily.

Answer: E

24. Supplemental criteria for diagnosing SIADH include:

1.    Normal thyroid and adrenal function

2.    Fractional sodium excretion >1%, fractional urea excretion >55%

3.    Clinical euvolemia

4.    No diuretic use in preceding days

5.    Abnormal water load test

A. 1, 2 B. 2, 4 C. 1, 3 D. 2, 5 E. 4, 5

Answer: D

25. True statements about etiology and prevalence of adrenal incidentalomas:

1.    Mild autonomous cortisol secretion (MACS) occurs in 20-30%

2.    Non-functioning tumors in 40-70%

3.    Primary hyperaldosteronism in 2-5%

4.    Myelolipoma in 10-20%

5.    Overt Cushing’s in 1-4%

A. 1, 3, 4, 5 B. 1, 2, 3, 5 C. 1, 2, 4, 5 D. 1, 4, 5 E. Only 3

Answer: B

26. Monitoring hypoparathyroidism includes:

1.    Serum calcium, magnesium, phosphate

2.    Ophthalmologic exam (risk of glaucoma)

3.    Renal evaluation (risk of nephrolithiasis)

4.    CNS imaging (risk of calcifications)

5.    Bone mineral density assessment

A. 1, 2 B. 1, 2, 3, 5 C. 2, 3, 4, 5 D. 1, 3, 4, 5 E. 2, 3

Answer: D

27. True statements about vitamin D:

1.    It belongs to steroid hormones

2.    1,25‑OH‑D3 is used to assess vitamin D status

3.    25‑OH‑D (calcifediol) is used to assess status

4.    Deficiency leads to increased PTH and phosphate

5.    Contraindicated in hyperparathyroidism

6.    Deficiency contributes to autoimmunity

A. 3, 5, 6 B. 2, 3 C. 1, 3, 4, 6 D. 2, 4, 5 E. 1, 3, 5

Answer: C

28. Highest risk of iatrogenic adrenal insufficiency is with:

A. Betamethasone and dexamethasone B. Methylprednisolone and prednisolone C. Prednisone and deflazacort D. Hydrocortisone and cortisone E. Methylprednisolone and triamcinolone

Answer: A

29. True statements about congenital hypothyroidism:

1.    It is the most common preventable cause of intellectual disability in newborns;

2.    Symptoms appear immediately after birth;

3.    Newborn screening includes TSH and/or T4 measurement;

4.    Treatment should start as soon as possible, ideally within the first 2 weeks of life;

5.    It is mostly caused by iodine deficiency.

A. 1, 3, 4 B. 2, 3, 5 C. 1, 2, 5 D. 3, 4, 5 E. 1, 4, 5

Answer: A

30. Which of the following are features of Grave’s disease?

1.    Diffuse goiter;

2.    Ophthalmopathy;

3.    Pretibial myxedema;

4.    Positive TSH receptor antibodies;

5.    High radioactive iodine uptake.

A. 1, 2, 4 B. 1, 3, 5 C. 2, 3, 4 D. 1, 2, 3, 4, 5 E. 3, 4, 5

Answer: D

31. In which conditions is thyroid peroxidase antibody (TPOAb) positivity commonly found?

1.    Hashimoto’s thyroiditis;

2.    Grave’s disease;

3.    Subacute thyroiditis;

4.    Postpartum thyroiditis;

5.    Toxic multinodular goiter.

A. 1, 2, 4 B. 2, 3, 5 C. 1, 3, 4 D. 1, 4 E. 3, 5

Answer: E

32. Which NET feature in the appendix alone warrants right hemicolectomy: A. G1 NET, 1.6 cm B. Located at the tip of appendix C. Lymphovascular invasion D. Germline MEN 1 mutation E. No mesoappendiceal invasion

Answer: C

33. A 35‑year‑old woman with Graves’ disease pregnant, TSH 1.0 mIU/L, FT3 and FT4 normal on 5 mg thiamazole; two miscarriages in history. Next steps:

1.    If low TRAb and no orbitopathy, consider tapering thiamazole with thyroid monitoring

2.    Always switch thiamazole to PTU in first trimester.

3.    If on PTU in first trimester, switch back to thiamazole in second and continue to term.

4.    Maintain FT4 in upper normal range during treatment.

5.    Measure TRAb at 18-22 weeks and again at 30-34 weeks if elevated.

A. 1, 2, 5 B. 2, 4, 5 C. 1, 2, 3, 4 D. 1, 4, 5 E. 1, 3, 4, 5

Answer: D

34. In combined estrogen-progestogen contraceptives, the progestogen lacking antiandrogenic activity is: A. Dienogest B. Drospirenone C. Levonorgestrel D. Cyproterone acetate E. Chlormadinone acetate

Answer: C

35. Marker recommended in endometriosis diagnostics: A. hCG B. CEA C. CA‑125 D. AFP E. No biochemical markers recommended

Answer:E

36. False statement about menstrual disorders: A. Primary amenorrhea is absence of menstruation before age 16. B. Secondary amenorrhea is 3‑month absence in previously menstruating woman. C. Algomenorrhea means painful menstruation. D. Oligomenorrhea: cycles longer than 32 days. E. Polymenorrhea: cycles shorter than 21 days.

Answer: B

37. In the modified Ferriman‑Gallwey scoring for hirsutism, which area is not assessed? A. Thigh B. Lower abdomen C. Forearm D. Lower back E. All of these are included.

Answer: C

38. Using Prader orchidometer, at what testicular volume does puberty begin? A. 2 mL B. 4 mL C. 6 mL D. 10 mL E. No set volume-based on pubic hair development

Answer: B

39. WHO category of menstrual disorders in anorexia nervosa: A. I B. II C. III D. IV E. V

Answer: A

40. True statement about hormone receptors in vaginal and perineal tissues: A. Estrogen receptors only in vaginal epithelium. B. Estrogen receptors least in vaginal apex, increasing toward vestibule. C. Estrogen receptors only pre‑menopause. D. Androgen receptors most in external genitalia. E. No androgen receptors in vagina.

Answer: D

41. True statement about macroprolactin in hyperprolactinemia: A. It’s more active than monomeric prolactin. B. Assessed via recovery after polyethylene glycol precipitation. C. It’s prolactin bound to glycerol. D. Hyperprolactinemia, regardless of cause, doesn’t affect reproduction. E. Occurs in 10-25% of women with hyperprolactinemia.

Answer: E

42. In newly discovered adrenal tumor, the first‑choice imaging to assess malignancy risk is: A. MRI B. Contrast CT C. High‑resolution single‑phase CT D. Adrenal ultrasound E. ¹⁸F‑FDG PET/CT

Answer: C

43. In a pregnant woman suspected of hypercortisolism in second trimester, which test should be ordered? A. 1 mg dexamethasone suppression test B. Morning serum cortisol C. Salivary cortisol D. Morning ACTH

Answer: C

44. A 66‑year‑old man has a 2 cm homogeneous left adrenal mass with <10 HU on CT, incidental finding. According to 2023 ESE guidelines, necessary hormonal tests include: A. 1 mg dexamethasone suppression test B. 24‑h urinary fractionated metanephrines C. Androstenedione measurement D. Aldosterone‑renin ratio (with normal potassium) E. All of the above

Answer: A

45. A 64‑year‑old man post-left lobectomy diagnosed with 14 mm papillary thyroid carcinoma, columnar cell subtype, no invasion. Recommended further treatment: A. Completion thyroidectomy + central neck dissection + RAI + TSH partially suppressive LT4 B. Completion thyroidectomy + central dissection, no RAI, LT4 replacement C. Whole-body scan and based on result, consider reoperation/RAI D. LT4 replacement doses only E. Fully suppressive LT4 therapy

Answer: A

46. A patient with two thyroid nodules (EU‑TIRADS‑PL‑3): right 24×14×24 mm (biopsy category IV), left 20×18×22 mm (biopsy category II). Does right nodule’s category IV result preclude treatment with ¹³¹I if the left is benign? A. Yes, category IV always indicates surgery. B. Yes, any category IV needs total thyroidectomy. C. No, if right nodule is autonomous on scintigraphy. D. No, if at least one lesion is autonomous. E. No, if 24‑h thyroid iodine uptake >30%.

Answer: C

47. A physician plans to perform a fine‑needle aspiration biopsy (FNAB) of a thyroid lesion in a patient with normal renal function who is chronically taking dabigatran. Should the treatment be modified before the biopsy? A. Yes-discontinue the drug ≥24 hours before the biopsy. B. Yes-discontinue the drug ≥48 hours before the biopsy. C. No, there is no need to discontinue the drug. D. Switch the patient to low‑molecular‑weight heparin before performing the biopsy. E. Postpone the biopsy until dabigatran therapy is completed.

Answer: A

48. Newborn screening for congenital hypothyroidism is performed on day 3 of life. An earlier measurement of TSH in a full‑term infant may be unreliable because in the first two days of life the TSH concentration is: A. physiologically elevated in all newborns due to a type‑2 allostatic phenomenon. B. still low due to underdeveloped negative feedback between TSH and thyroid hormones. C. falsely elevated because of maternal TSH that crosses the placenta into the infant’s circulation. D. falsely low due to high levels of placental gonadotropin in the infant’s blood. E. potentially falsely low due to TSH receptor antibodies transferred via the placenta into the infant’s circulation.

Answer: A

49. In a boy with Prader-Willi syndrome, delayed pubertal development is most commonly due to: A. hypothalamic dysfunction leading to GnRH deficiency. B. pituitary hypoplasia leading to LH and FSH deficiency. C. gonadal dysgenesis causing testosterone deficiency. D. elevated prolactin from pituitary stalk abnormalities, inhibiting the GnRH-LH/FSH-gonadal axis. E. androgen deficiency due to 11β-hydroxysteroid dehydrogenase type 1 deficiency, causing severely delayed bone age and later pubertal onset.

Answer: A

50. Low prolactin levels may occur in genetically conditioned combined deficiencies of TSH, prolactin, and GH (due to their common cell lineage), and also in patients treated with: A. newly diagnosed Parkinson’s disease. B. fluoxetine. C. proton‑pump inhibitors. D. aripiprazole. E. contraceptive medications.

Answer: D

51. The somatomedin generation test involves several days of administration of which substance? A. GHRH, with evaluation of IGF‑1 changes before and after the test. B. Somatostatin, with evaluation of IGF‑1 before and after. C. Growth hormone, with evaluation of IGF‑1 before and after. D. Macimorelin, with evaluation of IGF‑1 before and after. E. Pegvisomant, with evaluation of IGF‑1 before and after.

Answer: C

52. ACTH‑independent hypercortisolism (Cushing’s syndrome) may be caused by which of the following?

1.    Adrenocortical carcinoma (ACC)

2.    Primary bilateral macronodular adrenal hyperplasia (PBMAH)

3.    Cushing’s disease (pituitary ACTH‑secreting adenoma)

4.    Carney complex

5.    Small‑cell lung carcinoma

Answer: D

A. 1, 2

53. Regarding osteoporosis treatment in men, which is true? A. Testosterone replacement, even in deficient men, does not reduce fracture risk. B. Risedronate is the only bisphosphonate not registered for osteoporosis treatment in men. C. In men at very high fracture risk, romosozumab is recommended as first‑line therapy. D. In men, denosumab is recommended only as second‑line therapy. E. Unlike in women, treatment breaks from bisphosphonates are not recommended in men even if T‑score improves > -2.5 and no new fractures occur.

Answer: A

54. A 32‑year‑old female with a Bethesda VI thyroid lesion undergoes thyroidectomy and central lymph node dissection. Histopathology: papillary thyroid carcinoma, columnar cell variant, pT2N1aR0, maximum tumor size 24 mm, angioinvasion present, neuroinvasion absent, metastases in 6 of 11 central lymph nodes (<1 cm), postoperative ¹³¹I uptake only in the right thyroid bed. According to ATA/ESMO guidelines, what is the recurrence risk category? A. Low risk B. Intermediate risk C. High risk D. Very high risk E. Cannot be assigned with current data

Answer: B

55. In thyroid-associated orbitopathy, apart from decreased visual acuity, criteria for optic neuropathy include:

1.    Increased intraocular pressure

2.    Severity of proptosis

3.    Color vision impairment

4.    Visual field defects

5.    Optic disc pallor

A. 3, 4, 5 B. 1, 2, 3 C. 2, 3, 4, 5 D. 1, 3, 4, 5 E. 1, 2, 4, 5

Answer: A

55. According to the 2023 guidelines from the European Society of Endocrinology and ENSAT, a diagnosis of mild autonomous cortisol secretion (MACS) from an adrenal lesion requires:

1.    Presence of conditions associated with hypercortisolism (e.g., impaired glucose tolerance, diabetes, hypertension, osteoporosis)

2.    Overt Cushing’s phenotype (e.g., central obesity, plethoric face, wide red striae, spontaneous bruising, muscle wasting)

3.    Abnormal 1 mg dexamethasone suppression test (cortisol ≥ 50 nmol/L)

4.    Increased 24‑hour urinary free cortisol excretion

5.    Suppressed or low‑normal morning ACTH

A. 1, 3 B. 2, 3 C. 3, 5 D. 4, 5 E. 1, 3, 5

Answer: C

57. A 52‑year‑old woman has an incidentally discovered homogeneous left adrenal mass, 1.9 cm, density -8 HU on non‑contrast CT. According to the 2023 ESE/ENSAT guidelines, what is the next imaging step?

A. Contrast CT with washout evaluation B. Non‑contrast CT in 6-12 months C. Contrast‑enhanced MRI D. Non‑contrast MRI E. No further imaging needed

Answer: E

58. A 56‑year‑old man with Graves’ disease in remission undergoes pre‑coronary CT angiography labs: TSH 1.6 mIU/L (0.27-4.2), FT4 15.5 pmol/L (12-22). Per 2023 Polish Society of Endocrinology, the appropriate next step is:

A. Measure TRAb before imaging, with management based on TRAb levels. B. Repeat TSH post‑angiography only if hyperthyroid symptoms appear. C. Prophylactic tiamazole (20 mg daily starting day before CT, continue 14 days), with TSH check in 3-4 weeks. D. Prophylactic sodium perchlorate (600 mg before CT, then 3 × 300 mg for 7-14 days), with TSH check in 3-4 weeks. E. IV tiamazole (40 mg) before CT, with TSH check in 3-4 weeks.

Answer: B

59. Which is correct regarding treatment of hypoparathyroidism in pregnancy and lactation?

A. Active vitamin D analogs are FDA pregnancy category B. B. Placenta and breast tissue produce active vitamin D and PTH. C. Hypercalcemia in pregnancy increases uterine contractility and skeletal muscle excitability. D. During pregnancy/lactation, vitamin D analogs should be stopped or significantly reduced due to fetal risks. E. Calcium and vitamin D analog requirements may change during pregnancy.

Answer: E

60. Choose the true statement regarding pathogenesis and treatment of primary hypoparathyroidism:

A. PTH deficiency reduces renal 1α‑hydroxylase activity, limiting calcifediol synthesis. B. PTH deficiency reduces hepatic 25‑hydroxylation of vitamin D, thus calcifediol is needed. C. Treatment should maintain ionized or corrected calcium in the mid to upper reference range. D. Genetic causes are the most frequent cause of hypoparathyroidism. E. Calcitriol (1,25‑OH₂D) and alfacalcidol (1‑OH‑D) may be used interchangeably.

Answer: E

61 An ultrasound feature suggestive of benignity in EU‑TIRADS‑PL 3 or 4 thyroid nodules is:

A. Macrocalcifications B. Central vascular pattern C. Irregular thick halo D. Reduced compressibility E. Bright echoes with comet‑tail artifact

Answer:E

62. Select the true statement about pseudohypoparathyroidism:

A. PTH resistance may result from a GNAS gene defect. B. Resistance to TSH and gonadotropins (LH/FSH) may also be present. C. Biochemical profile: hypocalcemia, hyperphosphatemia, elevated PTH. D. Treatment of hypocalcemia can use active vitamin D analogs due to lower risk of hypercalciuria. E. All of the above.

Answer: E

63. Goals of chronic therapy in hypoparathyroidism include:

1.    Prevent symptoms of hypocalcemia

2.    Maintain phosphate in low‑normal range

3.    Prevent hypocalciuria

4.    Keep blood calcium slightly below normal

5.    Prevent ectopic renal calcifications

A. 1, 2, 3 B. 2, 3, 4 C. 2, 3, 5 D. 1, 4, 5 E. 1, 3, 5

Answer: D

64. Criteria for active surveillance in low‑risk papillary thyroid carcinoma (cT1aN0M0) include:

1.    Solitary lesion ≤1 cm

2.    Solitary lesion ≤2 cm

3.    Location near thyroid capsule

4.    No age criteria

5.    No lymph node metastases

A. 1, 5 B. 2, 5 C. 1, 4 D. 2, 3 E. 4, 5

Answer: A

65. True statement regarding pheochromocytoma:

A. All patients have hypertension. B. Surgical removal always cures hypertension. C. Small tumors are always hormonally inactive. D. Symptom severity always correlates with tumor size. E. None of the above.

Answer: E

66. Most likely MRI finding in a newly diagnosed patient with central diabetes insipidus is:

A. Pineal tumor B. Hyperintensity in cerebral cortex C. Cerebellar hypoplasia D. Hydrocephalus E. Loss of posterior pituitary bright spot on T1‑weighted images

Answer: E

67. Which drugs/substances cause decreased FT4 with normal TSH?

1.    Vitamin D

2.    Topiramate

3.    Glucocorticoids

4.    Amiodarone

5.    Estrogens

A. 1, 2 B. 2, 5 C. 1, 4 D. 2, 4 E. 3, 5

Answer: B

68. Postpartum thyroid follicular cell destruction with transient thyrotoxicosis is most often:

A. Autoimmune in origin B. Cause of severe hypothyroidism C. Due to iodine deficiency D. Due to uncontrolled iodine supplementation E. None of the above

Answer: A

69. TSH receptor antibodies (TRAb) should be measured in pregnant women with:

A. Hypothyroidism due to Hashimoto’s B. Hypothyroidism post‑thyroidectomy for nontoxic goiter C. No prenatal iodine prophylaxis D. Both A and B E. None of the above

Answer: E

70. In treating panhypopituitarism during pregnancy in a woman, the correct order of hormone replacement is: A. Recombinant prolactin → glucocorticoids → thyroid hormones → sex hormones → GH B. Glucocorticoids → thyroid hormones → sex hormones → GH C. Glucocorticoids → thyroid hormones → sex hormones D. Glucocorticoids → thyroid hormones E. Sex hormones → thyroid hormones → glucocorticoids → prolactin

Answer: D

71. For pregnant patients with known and properly treated adrenal insufficiency prior to conception: A. Discontinue glucocorticoids-they are teratogenic. B. Discontinue glucocorticoids but only in secondary adrenal insufficiency. C. Continue glucocorticoids, switching prednisone (if used) to hydrocortisone. D. Continue glucocorticoids, switching hydrocortisone to prednisone. E. Change the administration route from oral to IV or IM.

Answer: C

72. Which is not a symptom of adult growth hormone deficiency? A. Elevated triglycerides + decreased lean mass B. Increased total cholesterol + increased fat mass C. Decreased ejection fraction + decreased lean mass D. Decreased bone mineral density + elevated LDL fraction E. Decreased bone mineral density + elevated HDL fraction

Answer: E

73. In pregnancy, increased iodine requirements are: A. Because of increased fetal iodine degradation B. Met by potassium iodide supplements C. Only on iodine‑deficient regions D. Require milligram doses of iodine E. None of the above

Answer: B

74. Which false statement about thyroid hormone synthesis? A. Adequate iron intake is required. B. Reactive oxygen species from oxidative stress play important roles. C. Normal synthesis is independent of iodine supply. D. The thyroid produces much more T4 than T3. E. A large iodine dose acutely inhibits synthesis (Wolff-Chaikoff effect).

Answer: C

75. In pregnant women with newly diagnosed hypothyroidism, what is the appropriate treatment? A. Immediately start full replacement dose (even up to 200 µg/day) B. Start low‑dose levothyroxine and gradually increase until TSH <2.5 mIU/L C. Use T3 for rapid correction D. Use combined T4 and T3 for rapid correction E. Evaluate iron status first, then reassess before starting levothyroxine

Answer: A

76. In mild hypercalcemia, which medication does not cause elevated serum calcium? A. Hydrochlorothiazide B. Indapamide C. Calcifediol D. Denosumab E. Cholecalciferol

Answer: D

77. A 14‑week pregnant woman, previously treated for hyperthyroidism (etiology unknown), now has TSH 0.06 µIU/mL, FT4 17.8 pmol/L (9.01-19.05), FT3 5.16 pmol/L (2.63-5.7). What are initial recommendations? A. Start PTU and check labs in 4 weeks B. Start tiamazole and check labs in 4 weeks C. Block‑and‑replace with tiamazole + LT4, check labs in 4 weeks D. Cannot decide without knowing etiology E. Observe, checking labs every 2 weeks

Answer: E

78. Which is false regarding microcalcifications in thyroid nodules? A. They correspond to psammoma bodies typical of papillary carcinoma B. Round bright echoes ~1 mm in size C. Usually lack acoustic shadow D. Can be located within cystic areas of a lesion E. Their presence automatically classifies the nodule as EU‑TIRADS‑PL5

Answer: D

79. Which obesity drugs are currently approved in Poland for nondiabetic patients and EMA‑registered?

1.    Phentermine + topiramate

2.    Naltrexone + bupropion

3.    Orlistat

4.    Liraglutide

5.    Semaglutide

6.    Dulaglutide

7.    Tirzepatide A. 1, 3, 5, 6 B. 1, 2, 4, 7 C. 2, 3, 4, 5, 7 D. 2, 3, 4, 5, 6, 7 E. 2, 4, 7

Answer: C

80. In patients with obesity driven by emotional hunger, the 2024 PTH guidelines recommend starting with: A. Liraglutide B. Semaglutide C. Tirzepatide D. Phentermine + topiramate E. Naltrexone + bupropion

Answer: E

81. According to 2024 Polish obesity treatment guidelines, a successful pharmacotherapy is defined as: A. Any weight reduction B. Weight reduction that resolves or significantly improves ≥ one obesity‑related complication C. ≥5% weight reduction in 3 months at therapeutic dose D. ≥10% reduction in 3 months at therapeutic dose E. 5-10% reduction over 6 months at therapeutic dose

Answer: C

82. Maternal virilization during pregnancy with a virilized 46,XX newborn can be caused by:

1.    Lipoid congenital adrenal hyperplasia

2.    5α‑reductase deficiency

3.    Aromatase (P450) deficiency

4.    P450 oxidoreductase deficiency

5.    CYP17 deficiency A. 1, 2, 3 B. 3, 4, 5 C. 1, 2 D. 3, 4 E. 4, 5

Answer: D

83. In severe hypercalcemic crisis due to a PTH‑secreting parathyroid adenoma, intravenous hydration is combined with:

1.    Loop diuretics (e.g., furosemide)

2.    Thiazide diuretics (e.g., HCTZ)

3.    Bisphosphonates (e.g., pamidronate)

4.    Calcimimetics (cinacalcet) A. 1, 2, 3 B. 1, 2, 4 C. 1, 3, 4 D. 2, 3, 4 E. All of the above

84. In the differential diagnosis of hyponatremia, consider:

1.    Hypercortisolism (Cushing’s)

2.    11β‑HSD2 deficiency

3.    SIADH

4.    Cerebral salt‑wasting syndrome A. 1, 2, 3 B. 2, 3, 4 C. 1, 3 D. 2, 4 E. 3, 4

Answer: E

85. A boy with peripheral GnRH‑independent precocious puberty, café‑au‑lait spots, and fibrous bone dysplasia suggests: A. Albright’s osteodystrophy B. McCune-Albright syndrome C. Peutz-Jeghers syndrome D. Van Wyck-Grumbach syndrome E. Testotoxicosis

Answer: B

86. Gene mutations play a role in hormone receptor resistance syndromes such as:

1.    Albright’s osteodystrophy

2.    Nephrogenic diabetes insipidus

3.    McCune-Albright syndrome

4.    Rabson-Mendenhall syndrome A. 1, 2, 3 B. 1, 2, 4 C. 1, 3, 4 D. 2, 3, 4 E. All of the above

Answer: B

87. A 58‑year‑old woman on long‑term prednisone undergoes bone densitometry: T‑score -2.9 (spine), -3.2 (hip). Which treatment is not recommended? A. Alendronate 70 mg weekly B. Ibandronate 150 mg monthly C. Denosumab 60 mg subcut every 6 months D. IV zoledronic acid 5 mg E. Risedronate 35 mg weekly

Answer: B

88. A 34‑year‑old woman with T‑score -2.7 at L1-L4, no fractures, non-smoker, history of celiac disease. Next step: A. Start combined oral contraceptive to improve bone density B. Assess fracture risk using FRAX PL C. Evaluate calcium-phosphorus metabolism and vitamin D D. Start denosumab immediately E. Start IV zoledronic acid immediately

Answer: C

89. A 29‑year‑old woman with iatrogenic hypoparathyroidism: serum calcium 8.2 mg/dL, phosphorus 4.5 mg/dL, 25‑OHD 19 ng/mL, on calcium carbonate 2 g/d plus calcitriol 0.5 µg daily, asymptomatic. What should be done?

1.    Increase calcium dose

2.    Increase calcitriol dose

3.    Add cholecalciferol per general guidelines

4.    Add sevelamer to lower phosphorus

A. Only 2 B. Only 3 C. 1, 2 D. 2, 3 E. 2, 4

Answer: B

90. Post-thyroidectomy pathology shows NIFTP (non‑invasive follicular thyroid neoplasm with papillary‑like nuclear features). What is the most likely risk and management plan? A. Very low risk of recurrence/metastasis-observe only B. Very low risk-but in men, RAI recommended C. High lymph node metastasis risk-lymphadenectomy needed D. Risk of anaplastic transformation-lymphadenectomy + RAI needed E. Cannot determine risk

Answer: A

91. A 19‑year‑old with scheduled iodinated contrast CT has subclinical hypothyroidism. What is the appropriate management? A. Start levothyroxine and switch to MRI B. Start levothyroxine and proceed with CT as planned C. Start levothyroxine + tiamazole (block‑and‑replace) prior to CT D. Use sodium perchlorate prophylactically before CT E. Proceed with contrast CT without any changes

Answer: B

92. Most accurate method to assess metanephrines in pheochromocytoma diagnosis is: A. Urine spectrophotometry B. Urine HPLC C. Plasma LC‑MS/MS D. Plasma HPLC E. Plasma spectrophotometry

Answer: C

93. Which is not a cause of elevated SHBG? A. HCV infection B. HIV infection C. Carbamazepine use D. Mitotane use E. Hypothyroidism

Answer: E

94. In every case of incidentally discovered adrenal mass (incidentaloma), it is recommended to assess: A. 1 mg dexamethasone suppression test B. Aldosterone-renin ratio C. Testosterone levels D. Plasma metanephrines E. ACTH levels

Answer: A

95. A 46‑year‑old woman newly diagnosed with non‑classic congenital adrenal hyperplasia, with regular menses and mild hirsutism. Recommended management: A. Dexamethasone B. Hydrocortisone C. Combined oral contraceptive with drospirenone D. Combined oral contraceptive with dienogest E. No treatment

Answer: E

96. Patient with ENSAT stage II adrenocortical carcinoma, Ki‑67 12%, R1 resection. Next steps should include: A. Radiotherapy to surgical bed B. Radiotherapy + mitotane C. Chemotherapy (etoposide + cisplatin) D. Mitotane + etoposide + cisplatin E. Mitotane alone

Answer: B

97. A 30‑year‑old with Cushing’s disease after unsuccessful transsphenoidal surgery is treated with osilodrostat. Baseline: cortisol 8 AM 26.6 µg/dL, midnight 24.1; testosterone 0.49 (n ≤ 0.48); K⁺ 2.7; QTc 475 ms. After 3 months on 7 mg/day: cortisol 8 AM 12.1, midnight 12.6; testosterone 0.9; K⁺ 4.1; QTc 491. On antihypertensives and antidepressant. She reports worsening acne and hirsutism. BP 128/70. Which is the correct approach? A. Discontinue osilodrostat due to prolonged QT and switch to metyrapone. B. Discontinue osilodrostat due to androgen excess and switch to metyrapone. C. Increase osilodrostat dose. D. Decrease osilodrostat dose. E. Adjust other medications (non‑osilodrostat) that may prolong QT.

Answer: E

98. A 49‑year‑old with acromegaly, after failed pituitary surgery, was treated with lanreotide 120 mg/month for 6 months without normalization of IGF‑1, then switched to pegvisomant. After 3 months: GH 8.7 µg/L, IGF‑1 205 ng/mL (97-214), MRI unchanged. What should be done? A. Continue current therapy and MRI follow‑up B. Discontinue pegvisomant (no tumor regression nor GH/IGF‑1 reduction) and start pasireotide C. Discontinue pegvisomant and refer for reoperation D. Discontinue pegvisomant and refer for radiotherapy E. Add octreotide 40 mg/day to lower GH

Answer: A

99. A 26‑year‑old patient planning pregnancy presents with secondary amenorrhea and elevated prolactin levels without circadian rhythm, ranging from 170-210 ng/ml. Examination: mild galactorrhea; MRI shows 6 × 5 × 8 mm pituitary microadenoma. Other hormones: LH 0.7 IU/l, FSH 1.2 IU/l, TSH 0.7 mIU/l, FT4 16 pmol/l, morning cortisol 18 µg/dl, dex suppression cortisol 1.2 µg/dl, IGF‑1 268 ng/ml (97-214), GH OGTT: 0 min 1.75, 30 min 2.5, 60 min 2.8, 90 min 2.1, 120 min 1.9 ng/ml. What is the appropriate next step? A. Start dopamine agonist therapy only, at a dose that normalizes prolactin and allows for pregnancy. B. Start dopamine agonist and pasireotide; defer surgery until after pregnancy and childbirth. C. Start dopamine agonist and somatostatin analogue; defer surgery until after pregnancy and childbirth. D. Refer for transsphenoidal surgical resection. E. Use only somatostatin analogue and defer surgery until after pregnancy and childbirth.

Answer: D

100. A 68‑year‑old postmenopausal woman with osteoporosis (T‑score: femoral neck -2.9, L2‑L4 -2.6), history of humerus fracture 3 years ago and distal radius fracture 6 months ago, smokes 10 cigarettes/day. Receiving calcium carbonate and cholecalciferol. Labs: Ca 2.46 mmol/l, creatinine 0.8 mg/dl, eGFR 69 ml/min/1.73 m², ALT 18 IU/l, 25(OH)D 38 ng/ml, PTH 31 pg/ml, albumin 40 g/l. Meds: esomeprazole, ramipril. What is the correct statement about her treatment? A. Start bisphosphonates; if not tolerated, switch to denosumab. B. Use denosumab only, and switch to romosozumab only if ineffective. C. Start romosozumab within drug program, switch to denosumab after 12 months. D. Start romosozumab within drug program, switch to denosumab after max 24 months. E. Use denosumab as bisphosphonates are contraindicated, and romosozumab criteria are not met.

Answer: C

101. A 52‑year‑old woman with obesity (BMI 41), HTN, hypercholesterolemia, T2DM (HbA1c 8.1%) is planned for tirzepatide therapy. History: pulmonary embolism, DVT, scheduled for thyroidectomy (retrosternal goiter). Meds: metformin 3×850 mg, bisoprolol, valsartan, apixaban, indapamide, rosuvastatin. What is the correct statement? A. Caution with tirzepatide before thyroidectomy due to aspiration pneumonia risk under sedation. B. Therapy contraindicated due to high thromboembolic risk. C. Metformin dose should be reduced before tirzepatide. D. Monitor lipase during therapy and reduce/discontinue if elevated. E. Tirzepatide should not be used; semaglutide or liraglutide should be first-line.

Answer: A

102. Choose true statements about prolactinomas:

1.    TPIT is a specific transcription factor in histopathology;

2.    Surgery restores normoprolactinemia in 82% of microadenoma, 44% of macroadenoma cases;

3.    Prolactinomas make up ~50% of all pituitary adenomas in both sexes;

4.    Evidence suggests higher prolactinoma incidence in transgender women receiving hormone therapy;

5.    Genetic testing is indicated in patients <30 years with macroprolactinoma or family history. A. 1, 3. B. 2, 3, 5. C. 2, 4, 5. D. Only 1. E. Only 3.

Answer: B

103. Which endocrine disorder is associated with DAX1 gene mutation? A. Precocious puberty B. Gigantism C. Thyroid hormone resistance D. Cushing’s syndrome E. Congenital adrenal insufficiency with hypogonadotropic hypogonadism

Answer: E

104. Choose the false statement about DXA densitometry: A. Gold standard for osteoporosis diagnosis B. T‑score reflects standard deviation (SD) from peak bone mass C. T‑score > -1 SD = normal D. T‑score -1 to -2.5 SD = osteoporosis E. T‑score ≤ -2.5 SD plus fragility fracture = advanced osteoporosis

Answer: D

105. True statement about active vitamin D forms: A. Active forms (e.g., calcitriol) are justified in hypoparathyroidism because PTH deficiency and hyperphosphatemia impair liver conversion of 25‑OH‑D. B. Calcitriol dose requirement is same for all patients. C. Calcitriol is about twice as potent as alfacalcidol. D. Alfacalcidol and dihydrotachysterol do not require liver activation. E. Calcitriol requires liver activation.

Answer: C

106. True statements about bisphosphonates: A. Oral bisphosphonates are most commonly used and effective first‑line antiresorptives. B. IV bisphosphonates used when oral forms are contraindicated or after hip fracture. C. Their effectiveness depends on adequate 25(OH)D and calcium‑phosphate balance. D. A and C are true. E. A, B, and C are true.

Answer: E

107. TSH should not decrease in a patient: A. With asthma on theophylline B. With diabetes on biguanides C. With acromegaly on lanreotide D. On morphine for cancer pain E. Treated with hGH for GH deficiency

Answer: A

108. Which set of symptoms/diseases is associated with both primary hypothyroidism and somatotropinoma? A. Carpal tunnel, thickened skin, muscle weakness, galactorrhea B. Weight gain, voice change, hyperkalemia, angina C. Subcutaneous edema, HTN, galactorrhea, hirsutism D. Joint pain, low libido, constipation, valve disease, hypercalcemia E. Hyperlipidemia, thyroid cancer, hyponatremia, diabetes

Answer: E

109. A 32‑year‑old woman with schizophrenia on clozapine presents with galactorrhea and menstrual disorders. Labs: PRL 42 µg/L (N <20), PRL after metoclopramide stimulation: 278 µg/L. What next?

1.    Suggest psychiatrist switch clozapine to olanzapine and reassess PRL

2.    Continue clozapine and add cabergoline

3.    Diagnose functional hyperprolactinemia and observe

4.    Suspect prolactinoma - do pituitary MRI and visual field testing

5.    Dilute PRL sample 1:100 to rule out hook effect A. Only 1 B. Only 2 C. Only 3 D. 4, 5 E. None of the above

Answer: E

110. An obese 78‑year‑old man asks how much vitamin D to take. History: heart failure, COPD. Best approach:

1.    Measure serum vitamin D and dose accordingly

2.    If unavailable, use max recommended dose for age

3.    Supplement only during fall‑winter

4.    Supplement year‑round

5.    Calcefediol is first‑line in this case A. 1, 2, 3 B. 1, 2, 4 C. 1, 3, 5 D. 2, 3, 5 E. 2, 4, 5

Answear: B

111. Choose the true statement about mild autonomous cortisol secretion (MACS): A. First‑line treatment is steroidogenesis inhibitors B. Screen for HTN, diabetes, vertebral fractures C. Surgery is always required D. High risk of Cushing’s syndrome E. Diagnostic cortisol level in 1 mg DST is > 138 nmol/L (5.0 µg/dL)

Answer: B

112. Indicate the true statements regarding osteoporosis treatment:

1.    Teriparatide reduces hip fracture risk

2.    Risedronate reduces vertebral, hip, and nonvertebral fractures and helps in men

3.    Ibandronate is drug of choice in glucocorticoid‑induced fractures

4.    Teriparatide is approved for glucocorticoid‑induced osteoporosis

5.    SERMs reduce only spinal fractures and lower estrogen‑dependent breast cancer risk A. 1, 2, 3 B. 1, 4, 5 C. 2, 4, 5 D. 3, 4, 5 E. 1, 4

Answer: C

113. To assess ovarian reserve, one should: A. Measure LH and estradiol on day 7 of the cycle. B. Measure prolactin on day 12 of the cycle. C. Measure AMH levels regardless of cycle day. D. Measure progesterone on day 3 of the cycle. E. Perform ultrasound to calculate AFC (Antral Follicle Count) - number of antral follicles >10 mm.

Answer: C

114. Severe ovarian hyperstimulation syndrome (OHSS) is indicated by:

1.    Leukocytosis > 15,000/mm³;

2.    Hypoproteinemia;

3.    Decreased liver enzyme activity;

4.    Serum sodium > 135 mmol/l, potassium < 5 mmol/l;

5.    Serum creatinine > 1.5 mg/dl. A. 1, 2, 4 B. 2, 3, 5 C. 1, 2, 5 D. 3, 4 E. All of the above

Answer: C

115. Takotsubo cardiomyopathy can occur in the course of: A. Pheochromocytoma B. Hyperparathyroidism C. Cushing’s syndrome D. Hypothyroidism E. Primary adrenal insufficiency

Answer:A

116. The metabolic effects of glucocorticoids include:

1.    Stimulation of gluconeogenesis in the liver;

2.    Increased glucose utilization in peripheral tissues;

3.    Stimulation of protein synthesis in peripheral tissues, mainly muscles;

4.    Increased apoptosis of neutrophils;

5.    Stimulation of lipolysis with release of glycerol and fatty acids. A. 1, 5 B. 2, 3 C. 1, 4, 5 D. 2, 4, 5 E. All of the above

Answer: A

117. Ovarian steroidogenesis:

1.    Occurs in granulosa cells;

2.    Occurs in thecal cells;

3.    Accounts for the production of ~25% of circulating testosterone, 50% of androstenedione, and 20% of DHEA;

4.    Ceases completely after menopause, and ovaries no longer secrete androgens;

5.    Menopause directly affects testosterone levels. A. 1, 3, 5 B. 2, 3, 4 C. 2, 4, 5 D. 1, 2, 3 E. All of the above

Answer: D

118. Prolactin can be produced by:

1.    Lactotrophs (anterior pituitary cells);

2.    Endometrial lining (uterine mucosa);

3.    Placenta;

4.    Mammary glands;

5.    Cells of the reticuloendothelial system. A. Only 1 B. 2, 3 C. 1, 4, 5 D. 2, 3, 4 E. All of the above

Answer: E

119. Apparent mineralocorticoid excess is characterized by:

1.    Deficiency of 11β‑hydroxysteroid dehydrogenase;

2.    High cortisol levels;

3.    Virilization of genitalia;

4.    Low aldosterone levels;

5.    Low renin levels. A. 1, 4, 5 B. 2, 4, 5 C. 1, 3, 5 D. 1, 2, 5 E. 1, 2, 3, 4

Answer: A

120. Indicate true statements regarding the diagnosis of hyperandrogenism:

1.    In prepubertal boys, it leads to genital enlargement and increased muscle mass;

2.    The Ferriman‑Gallwey scale is used to assess the severity of hirsutism in 9 androgen‑dependent body areas;

3.    Hypertrichosis may be caused by hyperandrogenemia, and may be hereditary, idiopathic, or drug‑induced;

4.    In men, hyperandrogenemia can suppress gonadotropin secretion, reduce testosterone production, and impair spermatogenesis;

5.    Female‑pattern hair loss involves hair loss starting at the temples and visible at the crown.

A. 1, 2, 4 B. 2, 3, 5 C. 3, 4, 5 D. 1, 2, 5 E. All of the above

Answer: A

Appendix 1

Appendix 2 
